# A Consistent BGK Model with Velocity-Dependent Collision Frequency for Gas Mixtures 

**DOI:** 10.1007/s10955-021-02821-2

**Published:** 2021-09-12

**Authors:** J. Haack, C. Hauck, C. Klingenberg, M. Pirner, S. Warnecke

**Affiliations:** 1 Los Alamos National Laboratory, Los Alamos, NM 87545 USA; 2 Oak Ridge National Laboratory, 1 Bethel Valley Road, Bldg. 5700, Oak Ridge, TN 37831-6164 USA; 3 Universität Würzburg, Emil-Fischer-Str. 40, 97074 Würzburg, Germany

**Keywords:** Multi-fluid mixture, Kinetic model, BGK approximation, Plasma physics, Velocity-dependent collision frequency, Entropy minimization

## Abstract

We derive a multi-species BGK model with velocity-dependent collision frequency for a non-reactive, multi-component gas mixture. The model is derived by minimizing a weighted entropy under the constraint that the number of particles of each species, total momentum, and total energy are conserved. We prove that this minimization problem admits a unique solution for very general collision frequencies. Moreover, we prove that the model satisfies an H-Theorem and characterize the form of equilibrium.

## Introduction

In this paper, we present a BGK-type model for gas mixtures that, in the case of two species, takes the form

1 $$\begin{aligned} \begin{aligned} \partial _t f_1 + v \cdot \nabla _x f_1&= \nu _{11} (M_{11} - f_1) + \nu _{12} (M_{12}- f_1), \\ \partial _t f_2 + v \cdot \nabla _x f_2&=\nu _{22} (M_{22} - f_2) + \nu _{21} (M_{21}- f_2), \end{aligned} \end{aligned}$$

along with appropriate boundary and initial conditions. Here $f_1 = f_1(x,v,t)$ and $f_2 = f_2(x,v,t)$ are the number densities of species of mass $m_1$ and $m_2$, respectively, with respect to the phase space measure *dxdv*; $x\in {\mathbb {R}}^3$ is the position coordinate of phase space; $v\in {\mathbb {R}}^3$ is the velocity coordinate; and $t\ge 0$ is time. The relaxation operator on the right hand side of (1) involves target functions of the form

2 $$\begin{aligned} M_{kj} = \exp (m_k\lambda ^{kj}_0 + m_k \lambda ^{kj}_1 \cdot v + m_k\lambda ^{kj}_2 |v|^2), \end{aligned}$$

which depend on parameters $\lambda ^{kj}=(\lambda ^{kj}_0, \lambda ^{kj}_1, \lambda ^{kj}_2) \in {\mathbb {R}}\times {\mathbb {R}}^3 \times (-\infty ,0)$, and (non-negative) collision frequencies $\nu _{kj}$. These parameters depend implicitly on $f_1$ and $f_2$, and once specified, determine the BGK operator.

The purpose of the relaxation operator in (1) is to provide an approximation of the multi-species Boltzmann collision operator that is more computationally tractable, but still maintains important structural properties. In the single-species case, the original BGK model [2] serves this purpose. In particular, it has the same collision invariants as the Boltzmann operator (which lead to conservation of number, momentum, and energy) and it satisfies an H-Theorem. In the multi-species case, these requirements are not as straight-forward to satisfy, but it can be done. There are many BGK models for gas mixtures proposed in the literature [1, 5, 10, 12, 14–16, 23, 28], many of which satisfy these basic requirements and, in addition, are able to match some prescribed relaxation rates and/or transport coefficients that come from more complicated physics models or from experiment. Many of these approaches have been extended to accommodate ellipsoid statistical (ES-BGK) models, polyatomic molecules, chemical reactions or quantum gases; see for example [3, 4, 13, 24–27, 31].

A common feature of all the models mentioned above is that they only allow for collision frequencies which are independent of the microscopic velocity *v* of the particles [30]. However, the collision frequencies in principle should depend on the microscopic velocity, which is typically neglected for the reason of simplicity. In the case of neutral gases, velocity independent collision frequency leads to transport properties in the fluid regime that are inconsistent with the full kinetic collision operator, e.g., the Prandtl number. Models such as the ES-BGK model and the Shakov model make changes to the target Maxwellian to provide extra degrees of freedom to the system and correct this defect, but still retain the constant collision frequency assumption. Some attempts have been proposed to re-introduce velocity dependence in the case of variable hard spheres interactions for neutral gases [22], for which velocity-dependent collision frequencies are monotonically increasing and are well-defined.

In the case of Coulomb interactions, i.e. plasmas, the Boltzmann cross section is even more strongly dependent on the relative velocity of the particles, as particles with small relative velocity, or grazing collisions, are the dominant event. As a result, the cross section is near-singular in both the relative velocity and scattering angle variables, and approximating it with a single, constant value is likely to miss the rich underlying features of the grazing collision physics. Indeed, in a widely used model in hydrodynamic modeling of plasmas, based on a non-conservative but velocity-dependent BGK model [21], the exponent of the velocity component directly appears in the formulas for electrical and thermal conductivity. While we derive a conservative velocity dependent BGK model in this paper, we follow the spirit of [21] and define our velocity-dependent collision frequency in terms of the momentum transfer cross section, which results in a collision frequency that is decreasing in the limit of large relative velocities [20].

The velocity-dependent BGK model of this paper provides two advantages over more accurate Boltzmann and Fokker–Planck–Landau collision models from a computational perspective. Though computation of this model is not the focus of this paper, we mention these motivating concerns here for completeness; numerical discretization will be the subject of future work. First, like other BGK models it is amenable to implicit time discretization, which allows for time steps that exceed the frequently stiff collision frequency. While some proposed methods allow for time steps larger than the collision frequency in the Boltzmann or Landau–Fokker–Planck models [17], these methods are only weakly asymptotic preserving and it can be unclear without a priori knowledge of the size of the deviation from local Maxwellians. Furthermore, multispecies extension of these methods [18] penalize each species with a single relaxation operator rather than penalizing individual reaction pairs, which can lead to inaccuracies in cases where some collisional combinations are more important than others.

Another concern is the computational cost of a collision operator evaluation. The main cost of the BGK model presented here lies in the evaluation of the parameters in the target Maxwellians. When the collision frequencies are constant with respect to velocity, these parameters can be computed explicitly in terms of the given moments. In the velocity-dependent case, an optimization problem (similar to (8) and (15) presented below) must be solved numerically via a dual formulation. The main cost here lies in a Newton solve—specifically, the evaluation of the integrals that appear in the gradient and Hessian of the dual objective functions. In practice, the cost of the optimization will depend on the details of the implementation [34–37]. However, assuming that the velocity grid that is used to discretize the BGK equation in velocity space is also used to evaluate the integrals, then the method will scale like $O(N^3)$. For comparison, currently the fastest algorithms for the evaluating the Boltzmann collision operator are spectral methods. In three velocity dimensions, the complexity of evaluating specialized kernels is $O(M N^3 \log N)$ [32], while for general kernels, the complexity is $O(M N^4 \log N)$ [33]. Here *N* is the number of points in each dimension of the velocity space and *M* is the number of quadrature points needed to accurately approximate integrals over the unit sphere $S^2$ in ${\mathbb {R}}^3$. In practice, the size of *M* is problem dependent; and while $M \ll N^2$ is typical, it is not unusual to have $M \ge N$ [33]. Thus, while more expensive than the constant frequency case, the BGK model with velocity-dependent frequencies is still expected to be less expensive than evaluating the Boltzmann collision operator.

In this paper, we derive a model of the form (1) that allows for velocity-dependent collision frequencies. Our derivation includes as a by-product the single-species BGK model with velocity-dependent collision frequency that was proposed in [29]. We identify target functions that are consistent with the conservation laws for (1) and satisfy an entropy minimization principle. In particular, *intra-species* collisions (between the same species) should preserve mass, momentum, and energy within a species; that is,

3 $$\begin{aligned} \int m_k \nu _{kk} \begin{pmatrix} 1 \\ v \\ |v|^2 \end{pmatrix} (M_{kk} - f_k) dv = 0, \quad k \in \{1,2\}. \end{aligned}$$

Meanwhile *inter-species* collisions (between different species) should preserve the mass of each species, but only the combined momentum and energy of both; that is,

4 $$\begin{aligned} \begin{aligned} \int m_1 \nu _{12} (M_{12} - f_1) dv = 0,&\quad \int m_2 \nu _{21} (M_{21} - f_2) dv = 0 \\ \int m_1 \nu _{12} \begin{pmatrix} v \\ |v|^2 \end{pmatrix} (M_{12} - f_1) dv&+ \int m_2 \nu _{21} \begin{pmatrix} v \\ |v|^2 \end{pmatrix} (M_{21} - f_2)dv = 0. \end{aligned} \end{aligned}$$

When the collision frequencies are independent of *v*, the integrals in (3) and (4) can be computed explicitly, thereby providing relationships between the parameters $\lambda ^{kj}$ and the moments of $f_1$ and $f_2$ with respect to $\{1,v,|v|^2\}$. In the single-species case, this relationship defines the target function as the Maxwellian associated to *f*, while in the multi-species case, additional constraints must be imposed. However, when the collision frequencies depend on *v*, the aforementioned integrals are not always computable in closed form and the relationship between the parameters $\lambda ^{kj}$ and the moments of $f_1$ and $f_2$ with respect to $\{1,v,|v|^2\}$ cannot be written down analytically.

In spite of the difficulty of relating the target parameters to the moments of the kinetic distributions, the entropy minimization formulation can be still used to establish a unique set of parameters, under the conditions $\lambda _1^{12}= \lambda _1^{21}$ and $\lambda _2^{12} = \lambda _2^{21}$. We do so by adapting the strategy from [19] to fit the current setting. While a more abstract approach based solely on convex optimization tools can also be used [6], we follow [19] because it provides a more concrete connection to the application at hand. Our proof provides a rigorous justification for the target function used in [29] for the single species case. It also leads to an H-Theorem for the multi-species system (1).

The remainder of the paper is organized as follows. In Sect. 2, we motivate the choice of the target Maxwellians as solutions of minimization problems of the entropy under certain constraints. In Sect. 3, we prove existence and uniqueness of the minimization problems. In Sect. 4, we prove consistency of the model meaning that it satisfies the conservation properties, the H-Theorem and Maxwell distributions with equal mean velocity and temperature in equilibrium. In Sect. 5, we briefly summarize the straightforward extension to the case of *N* species, still with binary interactions.

## The Structure of the Target Functions

In this section, we motivate the form of the target functions in (2). It will be convenient in what follows to define the strictly convex function

5 $$\begin{aligned} h(z)= z \ln z -z, \quad z>0, \end{aligned}$$

and the vector-valued function

6 $$\begin{aligned} a^k(v) = \begin{pmatrix} a_0^k (v) \\ a_1^k (v) \\ a_2^k (v) \end{pmatrix} = \begin{pmatrix} m_k \\ m_k v \\ m_k |v|^2 \end{pmatrix}. \end{aligned}$$

Since *h* is convex and $h'(z) = \ln (z)$, it follows that

7 $$\begin{aligned} h(x) \ge h(y) + \ln (y)(x-y), \quad \forall ~ y,x \in {\mathbb {R}}^+. \end{aligned}$$

### The One Species Target Maxwellians

We seek a solution of the weighted entropy minimization problem

8 $$\begin{aligned} \min _{g \in \chi _k} \int \nu _{kk} h(g) dv, \quad k \in \{1,2\}, \end{aligned}$$

where

9 $$\begin{aligned} \chi _k= \left\{ g ~ \Big |~ g \ge 0,\, \nu _{kk} (1+ |v|^2) g \in L^1({\mathbb {R}}^3),\, \int \nu _{kk} a^k(v) (g - f_k) dv = 0 \right\} . \end{aligned}$$

The choice of the set $\chi _k$ ensures the conservation properties (3) for intra-species collisions. The motivation for weighting the usual objective by the collision frequencies in (8) is that the ansatz will take the form (2). Indeed, by standard optimization theory, any critical point $(M_{kk}, \lambda ^{kk})$ of the Lagrange functional $L_k :\chi _k \times {\mathbb {R}}^5 \rightarrow {\mathbb {R}}$, given by

10 $$\begin{aligned} L_k(g, \alpha ) = \int \nu _{kk} h(g) dv - \alpha \cdot \int \nu _{kk} a^k(v) (g - f_k) dv , \end{aligned}$$

satisfies the first-order optimality condition

11 $$\begin{aligned} \frac{\delta L_k}{\delta g} (M_{kk}, \lambda ^{kk}) = \nu _{kk} ( \ln M_{kk} - \lambda ^{kk} \cdot a^k(v) ) = 0, \end{aligned}$$

which implies then that

12 $$\begin{aligned} M_{kk} = \exp {( \lambda ^{kk} \cdot a^k)} = \exp {( m_k\lambda ^{kk}_0 + m_k \lambda ^{kk}_1 \cdot v + m_k\lambda ^{kk}_2 |v|^2)}. \end{aligned}$$

In Sect. 3.1, we prove in a rigorous way that there exists a unique function of the form (12) that satisfies these constraints.

#### Theorem 1

Suppose that there exists $\lambda ^{kk} \in {\mathbb {R}}\times {\mathbb {R}}^3 \times {\mathbb {R}}$ such that the function $M_{kk}$ given in (12) is an element of $\chi _k$. Then $M_{kk}$ is the unique minimizer of (8).

#### Proof

According to (7)

13 $$\begin{aligned} h(g) \ge h(M_{kk}) + \lambda ^{kk} \cdot a^k (g-M_{kk}), \end{aligned}$$

point-wise in *v*. Thus, because $\nu _{kk} \ge 0$, it follows that for all $g \in \chi _k$,

14 $$\begin{aligned} \int \nu _{kk} h(g) dv \ge \int \nu _{kk} h(M_{kk}) dv + \int \nu _{kk}\lambda ^{kk} \cdot a^k (g-M_{kk}) dv = \int \nu _{kk} h(M_{kk}) dv \end{aligned}$$

Hence $M_{kk}$ is a minimizer of (8), and uniqueness follows directly from the strict convexity of *h*. $\square$

### The Mixture Target Maxwellians

For interactions between species, we seek a solution of the weighted entropy minimization problem

15 $$\begin{aligned} \min _{g_{1}, g_{2} \in \chi _{12}} \int \nu _{12} h(g_{1}) dv + \int \nu _{21} h(g_{2}) dv , \end{aligned}$$

where

16 $$\begin{aligned} \begin{aligned} \chi _{12}= \Bigg \lbrace (g_{1}, g_{2})~\Big |&~ g_{1}, g_{2} >0,\, \nu _{12} (1+ |v|^2) g_{1},\, \nu _{21} (1+ |v|^2) g_{2} \in L^1({\mathbb {R}}^3),\\&\int m_1 \nu _{12} g_{1} dv = \int m_1 \nu _{12} f_1 dv, \quad \int m_2 \nu _{21} g_{2} dv = \int m_2 \nu _{21} f_2 dv, \\&\int m_1 \nu _{12} \begin{pmatrix} v \\ |v|^2 \end{pmatrix} (g_{1} - f_1) dv + \int m_2 \nu _{21} \begin{pmatrix} v \\ |v|^2 \end{pmatrix} (g_{2} - f_2) dv = 0 \Bigg \rbrace . \end{aligned} \end{aligned}$$

Here, $\chi _{12}$ is chosen such that the constraints (3) for inter-species collisions are satisfied. Similar to the case of intra-species collisions, we consider the Lagrange functional $L :\chi \times {\mathbb {R}} \times {\mathbb {R}} \times {\mathbb {R}}^3 \times {\mathbb {R}} \rightarrow {\mathbb {R}}$

17 $$\begin{aligned} \begin{aligned} L(g_1, g_2, \alpha _0^1,\alpha _0^2, \alpha _1, \alpha _2) =&\int \nu _{12} h(g_{1}) dv + \int \nu _{21} h(g_{2}) dv \\&-\alpha _0^1 \int m_1 \nu _{12} (g_{1} - f_1 )dv - \alpha _0^2 \int m_2 \nu _{21} (g_{2} - f_2 )dv \\&- \alpha _1 \cdot \left( \int m_1 \nu _{12} v (g_1 - f_1) dv + \int m_2 \nu _{21} v (g_2 - f_2) dv \right) \\&- \alpha _2 \left( \int m_1 \nu _{12} |v|^2 (g_1 - f_1) dv + \int m_2 \nu _{21} |v|^2 (g_2 - f_2) dv \right) .\\ \end{aligned} \end{aligned}$$

Any critical point $(M_{12}, M_{21}, \lambda _0^1,\lambda _0^2, \lambda _1, \lambda _2)$ of *L* satisfies the first-order optimality conditions

18 $$\begin{aligned} \frac{\delta L}{\delta g_1}(M_{12}, M_{21}, \lambda _0^1,\lambda _0^2, \lambda _1, \lambda _2) = \nu _{12} ( \ln M_{12} - \lambda ^{12} \cdot a^1(v) ) =0, \end{aligned}$$

19 $$\begin{aligned} \frac{\delta L}{\delta g_2}(M_{12}, M_{21}, \lambda _0^1,\lambda _0^2, \lambda _1, \lambda _2) = \nu _{21} ( \ln M_{21} - \lambda ^{21} \cdot a^2(v) ) =0, \end{aligned}$$

where $\lambda ^{12} = (\lambda _0^1, \lambda _1, \lambda _2)$ and $\lambda ^{21} = (\lambda _0^2, \lambda _1, \lambda _2)$. Therefore

20 $$\begin{aligned} M_{12}&= \exp (\lambda ^{12} \cdot a^1(v)) = \exp \left( m_1 \lambda _0^{12} + m_1 \lambda _1 \cdot v + m_1 \lambda _2 |v|^2 \right) \end{aligned}$$

21 $$\begin{aligned} M_{21}&= \exp (\lambda ^{21} \cdot a^2(v)) = \exp \left( m_2 \lambda _0^{21} + m_2 \lambda _1 \cdot v + m_2 \lambda _2 |v|^2 \right) . \end{aligned}$$

Since we only require conservation of the *combined* momentum and kinetic energy, there is only one Lagrange multiplier for the momentum constraint and one Lagrange multiplier for the energy constraint. Therefore, $\lambda _1^{12} = \lambda _1^{21}$ and $\lambda _2^{12} = \lambda _2^{21}$ in (2). When the collision frequency is constant, this restriction is the same as the one used in [15], but more restrictive than the model in [23].

In the next section, we prove the existence of functions of the form (2) that satisfy the constraints in (3) and (4). As in the single species case, it follows that these functions are unique minimizer of the corresponding minimization problem.

#### Theorem 2

Assume that there exist $\lambda _0^{12} \in {\mathbb {R}}$, $\lambda _0^{21} \in {\mathbb {R}}$, $\lambda _1^{12} = \lambda _1^{21} \in {\mathbb {R}}^3$, and $\lambda _2^{12} = \lambda _2^{21} \in {\mathbb {R}}$ such that the pair $(M_{12},M_{21})$, where $M_{kj}$ is defined in (2), is an element of $\chi _{12}$. Then $(M_{12},M_{21})$ is the unique minimizer of (15).

#### Proof

According to (7)

22 $$\begin{aligned} h(g) \ge h(M_{kj}) + \lambda ^{kj} \cdot a^k (g-M_{kj}), \end{aligned}$$

point-wise in *v*, for any measurable function *g* and $k,j \in \{1,2\}$. Therefore, since $\nu _{kj} \ge 0$, it follows that for any measureable functions $g_1$ and $g_2$,

23 $$\begin{aligned} \int \nu _{12}h(g_1) dv + \int \nu _{21} h(g_2) dv&\ge \int \nu _{12} h(M_{12}) dv + \int \nu _{21} h(M_{21})dv \nonumber \\&\quad + \lambda ^{12} \cdot \int \nu _{12} a^1 (g_1-M_{12})dv + \lambda ^{21} \cdot \int \nu _{21} a^2 (g_2-M_{21})dv. \end{aligned}$$

Since $$\lambda _1^{12}=\lambda _1^{21}$$ and $$\lambda _2^{12}=\lambda _2^{21}$$,

24 $$\begin{aligned} \begin{aligned} \lambda ^{12} \cdot \int \nu _{12} a^1&(g_1-M_{12}) dv + \lambda ^{21} \cdot \int \nu _{21} a^2 (g_2-M_{21})dv \\&= \lambda _0^{12} \int \nu _{12} m_1 (g_1-M_{12})dv + \lambda _0^{21} \int \nu _{21} m_2 (g_2-M_{21})dv \\&\quad + \lambda _1^{12} \cdot \left( \int \nu _{12} m_1 v (g_1-M_{12}) dv + \int \nu _{21} m_2 v (g_2-M_{21}) dv \right) \\&\quad + \lambda _2^{12} \left( \int \nu _{12} m_1 |v|^2 (g_1-M_{12}) dv + \int \nu _{21} m_2 |v|^2 (g_2-M_{21}) dv \right) . \end{aligned} \end{aligned}$$

If $(g_1,g_2)$ and $(M_{12},M_{21})$ are elements of $\chi _{12}$, then the constraints in (16) imply that each of the terms above is zero. In such cases, (23) reduces

25 $$\begin{aligned} \int \nu _{12}h(g_1) dv + \int \nu _{21} h(g_2) dv \ge \int \nu _{12} h(M_{12}) dv + \int \nu _{21} h(M_{21})dv, \end{aligned}$$

which shows that $(M_{12},M_{21})$ solves (15). Since the collision frequencies $\nu _{12}$ and $\nu _{21}$ are non-negative and *h* is strictly convex, it follows that this solution is unique. $\square$

## Existence and Uniqueness of the Target Maxwellians

In this section, we prove the existence of the multipliers $$\lambda ^{11}$$, $$\lambda ^{22}$$, $$\lambda ^{12}$$ and $$\lambda ^{21}$$ such that the single-species targets $$M_{11}$$ and $$ M_{22}$$ satisfy (3) and the mixture targets $$M_{12}$$ and $$M_{21}$$ satisfy (4). We follow closey the strategy laid out in [19], although some variations will be needed to account for the velocity-dependent collision frequencies and the mixture targets.

Throughout the paper, we denote a distribution function of exponential form by

26 $$\begin{aligned} \exp ^k_{\lambda }(v):= \exp (\lambda \cdot a^k(v)), \quad \lambda =(\lambda _0, \lambda _1, \lambda _2) \in {\mathbb {R}}^5. \end{aligned}$$

and let

27 $$\begin{aligned} D_{kj}= \lbrace g \ge 0 \mid \nu _{kj}(1+ |v|^2) g \in L^1({\mathbb {R}}^3), \, g \not \equiv 0 \rbrace , \quad \varLambda ^{kj} = \lbrace \lambda \in {\mathbb {R}}^5 \mid \exp ^k_{\lambda } \in D_{kj} \rbrace . \end{aligned}$$

For any $g \in D_{kj}$ the moment map $\mu ^{kj}$ is given by

28 $$\begin{aligned} \mu ^{kj}(g) = \begin{pmatrix} \mu ^{kj}_0 \\ \mu ^{kj}_1 \\ \mu ^{kj}_2 \end{pmatrix}(g) = \int \nu _{kj} a^k(v) g(v) dv . \end{aligned}$$

We make the following assumptions about the collision frequencies.

### Assumption 1

Each frequency $\nu _{kj}$ is strictly positive and defined such that

29 $$\begin{aligned} \varLambda := \varLambda ^{kj} = \{\lambda \mid \exp ^k_{\lambda } \in L^1({\mathbb {R}}^3) \} = \lbrace \lambda \in {\mathbb {R}}^5 \mid \lambda _2 <0 \rbrace \end{aligned}$$

is independent of *k* and *j*.

Roughly speaking, these assumptions are used to ensure integrability properties that are satisfied when the collision frequencies are independent of the velocity. They are used in the technical details of the proofs below, but are in practice satisfied by many realistic frequency models.

### Target Functions for Intra-species Collisions

We start the intra-species case; that is, for $k \in \{1,2\}$, we show the existence of multiplier $\lambda ^{kk}$ such that $M_{kk}$ satisfies (3). The basic idea is to show that the dual function

30 $$\begin{aligned} z(\lambda ; \rho ) = \mu ^{kk}_0(\exp ^k_{\lambda }) - \lambda \cdot \rho \end{aligned}$$

is differentiable and attains its minimum on $\varLambda$ for any $\rho \in \mu ^{kk}(D_{kk})$. Then the necessary condition for an extremum in $\varLambda$ yields

31 $$\begin{aligned} 0= \nabla _{\lambda } z(\lambda ^{kk}) = \int \nu _{kk}(v) a^k(v) \exp (\lambda ^{kk} \cdot a^k(v)) dv - \rho , \end{aligned}$$

which gives $$\rho = \mu ^{kk}(\exp ^k_{\lambda ^{kk}})$$.

#### Lemma 1

The function *z* is strictly convex and twice Fréchet differentiable on $\varLambda$.

#### Proof

It is sufficient to prove that $\phi (\lambda ) = \mu ^{kk}_0(\exp ^k_{\lambda })$ is strictly convex and twice Fréchet differentiable, with first derivative $D\phi (\lambda )= \mu ^{kk}(\exp ^k_{\lambda })$ and Hessian $H\phi (\lambda )= \int a^k(v) \otimes a^k(v) \exp _{\lambda }^k dv$. Convexity following immediately from convexity of the exponential function and linearity of the integral. Specifically, given $\lambda ^{(1)}$, $\lambda ^{(2)}$ and two positive scalars $\theta _1, \theta _2$ such that $\theta _1 + \theta _2 = 1$, it follows that $\exp ^k_{\theta _1 \lambda ^{(1)} + \theta _2 \lambda ^{(2)}} \le \theta _1 \exp ^k_{\lambda ^{(1)}} + \theta _2 \exp ^k_{\lambda ^{(2)}}$. Hence

32 $$\begin{aligned} \begin{aligned} \phi (\theta _1 \lambda ^{(1)} + \theta _2 \lambda ^{(2)} ) = \mu ^{kk}_0(\exp ^k_{\theta _1 \lambda ^{(1)} + \theta _2 \lambda ^{(2)}})&\le \mu ^{kk}_0(\theta _1 \exp ^k_{\lambda ^{(1)}} + \theta _2 \exp ^k_{\lambda ^{(2)}}) \\&= \theta _1\phi ( \lambda ^{(1)}) + \theta _2 \phi (\lambda ^{(2)} ). \end{aligned} \end{aligned}$$

For any nonzero $\delta \in {\mathbb {R}}^5$

33 $$\begin{aligned} \frac{ \phi (\lambda + \delta )- \phi (\lambda ) - D \phi (\lambda ) \cdot \delta }{|\delta |} = \int f_{\delta }(v) dv, \end{aligned}$$

where

34 $$\begin{aligned} f_{\delta }(v) = \nu _{kk}(v) \exp ^k_\lambda (v) \left( \frac{\exp ^k_\delta (v) - 1 - a^k(v) \cdot \delta }{|\delta |} \right) . \end{aligned}$$

A Taylor series expansion shows that

35 $$\begin{aligned} \begin{aligned} \left| \frac{\exp ^k_\delta (v) - 1 - \delta \cdot a^k(v)}{|\delta |}\right| =&\left| \sum _{n=2}^{\infty } \frac{(\delta \cdot a^k(v))^n}{n!} \frac{1}{|\delta |} \right| \le |a^k(v)| \sum _{n=1}^{\infty } \frac{|\delta \cdot a^k(v)|^n}{n!} \\ \le&|a^k(v)| \exp (|\delta \cdot a^k(v)|) \end{aligned} \end{aligned}$$

Therefore $$f_{\delta }(v) \le \exp ^k_{\lambda /2}(v) g_{\delta }(v)$$, where

36 $$\begin{aligned} \begin{aligned} g_{\delta }(v)&:= \nu _{kk}(v) |a^k(v)| \exp ^k_{\lambda /2}(v) \exp (|\delta \cdot a^k(v)|) \\ {}&\le \nu _{kk}(v) |a^k(v)| \left( \exp ^k_{\lambda /2+\delta }(v) + \exp ^k_{\lambda /2-\delta }(v) \right) . \end{aligned} \end{aligned}$$

Because $\varLambda$ is open, for $|\delta |$ sufficiently small, $\exp _{\lambda /2+\delta }(v)$ and $\exp _{\lambda /2-\delta }(v)$ are elements of $D^{kk}$, in which case $g_{\delta }$ is integrable. Moreover, $\exp _{\lambda /2}$ is bounded. Hence $f_{\delta }$ is bounded above by an integrable function and the dominated convergence theorem gives

37 $$\begin{aligned} \lim _{\delta \rightarrow 0} \int f_{\delta }(v) dv = \int \lim _{\delta \rightarrow 0} f_{\delta }(v) dv =0. \end{aligned}$$

The existence of the Hessian can be proven in an analogous way. $\square$

#### Lemma 2

For fixed ${\lambda } \in \varLambda$, $\xi \in S^5$, and $\rho \in \mu ^{kk}(D_{kk})$, the function

38 $$\begin{aligned} z_\xi (s) = z({\lambda } +s \xi ;\rho ) \end{aligned}$$

attains its unique minimum in the open interval

39 $$\begin{aligned} I(\xi , {\lambda }):= (- s_\mathrm{{b}}(- \xi , {\lambda }), s_\mathrm{{b}}(\xi , {\lambda })) \end{aligned}$$

where

$$\begin{aligned} s_\mathrm{{b}}(\xi , {\lambda }):= \sup \lbrace s: {\lambda } + s \xi \in \varLambda \rbrace \end{aligned}$$

takes the value $+ \infty$ if the boundary $\partial \varLambda$ is not met in the direction $\xi .$

#### Proof

The fact that *z* is strictly convex and differentiable with respect to $\lambda$ implies that $z_\xi$ is strictly convex and differentiable with respect to *s*. Hence it attains a unique minimum on the closure of $I(\xi , {\lambda })$.

We now show that $z_{\xi }$ cannot attain its minimum on the boundary of $I(\xi , {\lambda })$. Suppose first that $s_\mathrm{{b}}(\xi ,{\lambda }) < \infty$. According to Assumption 1, ${\lambda } + s_\mathrm{{b}}(\xi , {\lambda }) \xi \not \in \varLambda$. Hence by Fatou’s Lemma,

40 $$\begin{aligned} \lim _{s \rightarrow s_\mathrm{{b}}(\xi , {\lambda })} \int \nu _{kk} \exp ^k_{{\lambda } + s \xi } dv \ge \int \nu _{kk} \exp ^k_{{\lambda } + s_\mathrm{{b}}(\xi , {\lambda }) \xi } dv = \infty \end{aligned}$$

which implies that $\lim _{s \rightarrow s_\mathrm{{b}}(\xi , {\lambda })}z_\xi (s) = + \infty$.

Suppose now that $s_\mathrm{{b}}(\xi , {\lambda })= \infty$. There are two cases:

**Case 1:** $\xi \cdot a^{k}(v) \le 0$ for a.e. $v \in {\mathbb {R}}^3$. Since $\rho \in \mu ^{kk}(D_{kk})$, there exists $g \in D_{kk}$ such that $\rho = \mu ^{kk}(g)$. By definition, *g* is not identically zero and by Assumption 1$\nu _{kk}>0$. Thus the set
  41 $$\begin{aligned} \varOmega := \lbrace v \in {\mathbb {R}}^3 \mid \xi \cdot a^{k}(v) <0\rbrace \cap \lbrace v \in {\mathbb {R}}^3 \mid \nu _{kk}(v) g(v) >0 \rbrace \end{aligned}$$
  has positive measure. Hence
  42 $$\begin{aligned} \xi \cdot \rho = \xi \cdot \mu ^{kk}(g) = \int \nu _{kk}(v) \xi \cdot a^{k}(v) g(v) dv < 0 \end{aligned}$$
  so that
  43 $$\begin{aligned} \lim _{s \rightarrow \infty } z_{\xi }(s) = \lim _{s \rightarrow \infty } \int \exp ^{k}_{\lambda + s \xi } dv - (\lambda + s \xi ) \cdot \rho \ge \lim _{s \rightarrow \infty } - (\lambda + s \xi ) \cdot \rho = \infty . \end{aligned}$$
**Case 2:** $\lbrace v \in {\mathbb {R}}^3: \xi \cdot a^k(v)>0 \rbrace$ has positive measure. Then there exists an $\varepsilon >0$ such that $B=\lbrace v \in {\mathbb {R}}^3: \xi \cdot a^k(v) \ge \varepsilon \rbrace$ has positive measure. Hence
  44 $$\begin{aligned} \lim _{s \rightarrow \infty } z_{\xi }(s) \ge \lim _{s \rightarrow \infty } \left( \left( \int _B \nu _{kk}(v) \exp ^k_{{\lambda }} dv \right) \exp (s \varepsilon ) - ({\lambda }+ s \xi ) \rho \right) = \infty \end{aligned}$$
  due to exponential growth in *s*.$\square$

#### Theorem 3

For any $\rho \in \mu ^{kk}(D_{kk})$, the function $z(\cdot ;\rho )$ has a unique minimizer $\lambda ^* \in \varLambda$.

#### Proof

Let $\lbrace \lambda ^{(\ell )} \rbrace _{\ell =0}^{\infty }$ be an infimizing sequence such that $z(\lambda ^{(\ell )}) \rightarrow z_*$, where

$$\begin{aligned} z_* = \inf _{\lambda \in \varLambda } z(\lambda ). \end{aligned}$$

Let $d^{(\ell )}=\lambda ^{(\ell )}-\lambda ^{(0)}; ~ \ell \ge 1$ and set $\xi ^{(\ell )} = {d^{(\ell )}}/{||d^{(\ell )}||}$. Then $\xi ^{(\ell )} \rightarrow \xi ^* \in S^{4}$ possibly via a subsequence, because $S^{4}$ is compact. For any $\xi \in S^{4},$ let $s_*(\xi )= \arg \min _{s \in {\mathbb {R}}} z(\lambda ^{(0)} + s \xi ; \rho )$ which, according to Lemma 2, is well-defined. Because *z* is strictly convex and twice differentiable,

$$\begin{aligned}&(i) \quad g(\xi , s):= \partial _s z(\lambda ^{(0)} + s \xi ; \rho )=0 \quad \text {if and only if} \quad s=s_*(\xi ) \\&(ii) \quad \partial _s g(\xi , s) >0 \end{aligned}$$

Thus the implicit function theorem implies that $s_*$ is a $C^1$ function in a neighbourhood $N(\xi _*) \subset \varLambda$ that satisfies

45 $$\begin{aligned} g(\xi , s_*(\xi )) =0. \end{aligned}$$

Let $\ell _*$ be large enough that $\xi ^{(\ell )} \in N(\xi _*)$ for all $\ell \ge \ell _*$. Then

46 $$\begin{aligned} z(\lambda ^{(\ell )}; \rho ) = z(\lambda ^{(0)} + d^{(\ell )}; \rho ) = z(\lambda ^{(0)} + ||d^{(\ell )}|| \xi ^{(\ell )}; \rho ) \ge z(\lambda ^{(0)} + s_*(\xi ^{(\ell )}) \xi ^{(\ell )}; \rho ). \end{aligned}$$

Because $s_*$ is continuous on $N(\xi _*)$ the sequence $s_*(\xi ^{(\ell )}) \rightarrow s_*(\xi ^{*})$ with $|s_*(\xi ^{*})|< \infty$. Moreover, since *z* is continuous

47 $$\begin{aligned} z_* = \lim _{\ell \rightarrow \infty } z(\lambda ^{(\ell )}) \ge \lim _{\ell \rightarrow \infty } z(\lambda ^{(0)} + s_*(\xi ^{(\ell )}) \xi ^{(\ell )}) = z(\lambda ^{(0)} + s_*(\xi ^{*}) \xi ^*) \ge z_*, \end{aligned}$$

where first inequality follows from (46). Hence the infimum is attained at $\lambda _* = \lambda ^{(0)} + s_*(\xi ^{*}) \xi _* \in \varLambda .$

#### Corollary 1

Given any $f_k \in D_{kk}$, there exists a unique multiplier $\lambda ^{kk}$ such that $M^{kk}$ given by (2) solves (8).

#### Proof

Let $\rho _k = \mu ^{kk}(f_k)$. According to Theorem 3, $z(\cdot ,\rho _k)$ has a unique minimizer in $\varLambda$, which we denote by $\lambda ^{kk}$. By Lemma 1, $z(\cdot ,\rho _k)$ is also differentiable, so the first-order optimality condition (31) implies that $\rho _k = \mu ^{kk}(\exp _{\lambda ^{kk}})$. The result then follows from Theorem 1. $\square$

### Target Functions for Inter-species Collisions

In this section we show the existence of the multipliers $\lambda ^{12} = (\lambda _0^{12}, \lambda _1^{12}, \lambda _2^{12}) \in {\mathbb {R}} \times {\mathbb {R}}^3 \times {\mathbb {R}}$ and $\lambda ^{21} =(\lambda _0^{21}, \lambda _1^{21}, \lambda _2^{21}) \in {\mathbb {R}} \times {\mathbb {R}}^3 \times {\mathbb {R}}$ such that $\lambda _1^{12}=\lambda _1^{21}$, $\lambda _2^{12}=\lambda _2^{21}$, and $M_{12}$ and $M_{21}$ satisfy (4). Denote

48 $$\begin{aligned} {\lambda } =(\lambda ^{1}_0, \lambda ^{2}_0, \lambda _1, \lambda _2) \quad {\lambda }^{1} = (\lambda ^{1}_0, \lambda _1, \lambda _2) \quad \lambda ^{2} = (\lambda ^{2}_0, \lambda _1, \lambda _2) \end{aligned}$$

and use this notation for other vectors when appropriate.

Given $$g_1,g_2 \in D$$, let

49 $$\begin{aligned} {\bar{\mu }}(g_1,g_2) = \begin{pmatrix} \mu _0^{12}(g_1) \\ \mu _0^{21}(g_2) \\ \mu _1^{12}(g_1) + \mu _1^{21}(g_2) \\ \mu _2^{12}(g_1) + \mu _2^{21}(g_2) \end{pmatrix}. \end{aligned}$$

For any ${{\bar{\rho }}} \in {\bar{\mu }}(D_{12} \times D_{21})$, introduce the dual function

50 $$\begin{aligned} {\bar{z}}(\lambda ; {{\bar{\rho }}}) = \mu ^{12}_0(\exp ^1_{\lambda ^1}) + \mu ^{21}_0(\exp ^2_{\lambda ^2}) - \lambda \cdot {{\bar{\rho }}}. \end{aligned}$$

Similar to the intra-species case, our goal is to show that for any such ${{\bar{\rho }}}$, $z(\lambda ; {{\bar{\rho }}})$ attains its minimum on

51 $$\begin{aligned} {\bar{\varLambda }}= \lbrace \lambda \in {\mathbb {R}}^6: \lambda ^{1}, \lambda ^{2} \in \varLambda \rbrace . \end{aligned}$$

Then the necessary first-order condition for a minimum at $\lambda$

52 $$\begin{aligned} 0= \nabla _{\lambda } z(\lambda ;{{\bar{\rho }}}) = {\bar{\mu }}({\exp ^1_{\lambda ^{1}}(v), \exp ^2_{\lambda ^{2}}(v)}) - {{\bar{\rho }}}, \end{aligned}$$

which recovers the required constraints in (4), if we set $\lambda ^{12}=\lambda ^{1}$ and $\lambda ^{21}=\lambda ^{2}$.

#### Lemma 3

The function ${\bar{z}}$ defined in (50) is strictly convex and twice Fréchet differentiable on ${{\bar{\varLambda }}}$.

#### Proof

Differentiability of the ${{\bar{z}}}$ can be deduced as in the intra-species case by simply following the arguments of Lemma 1. We skip these details. Convexity also follows in a similar way. Let ${\bar{\phi }}(\lambda ) = \mu ^{12}_0(\exp ^1_{\lambda ^1}) + \mu ^{21}_0(\exp ^2_{\lambda ^2})$, then convexity of the exponential function implies that for any $\theta \in (0,1)$, $\lambda \in {\bar{\varLambda }}$, and $\beta \in {\bar{\varLambda }}$,

53 $$\begin{aligned} \begin{aligned} {\bar{\phi }}(\theta \lambda ) + {\bar{\phi }}((1-\theta ) \beta )&= \mu ^{12}_0(\exp ^1_{\theta \lambda ^1 + (1-\theta ) \beta ^1}) + \mu ^{21}_0(\exp ^2_{\theta \lambda ^2 + (1-\theta ) \beta ^2}) \\&\le \mu ^{12}_0(\theta \exp ^1_{\lambda ^1} + (1-\theta ) \exp ^1_{\beta ^1}) + \mu ^{21}_0(\theta \exp ^2_{\lambda ^2} + (1-\theta ) \exp ^2_{\beta ^2}) \\&= \theta {\bar{\phi }}(\lambda ) + (1-\theta ) {\bar{\phi }}(\beta ) \end{aligned} \end{aligned}$$

Thus ${\bar{\phi }}$ is strictly convex, as is ${\bar{z}}$, since the two functions differ only by a linear term. $\square$

#### Lemma 4

For ${\lambda } \in {{\bar{\varLambda }}} , \xi \in S^5$, and ${{\bar{\rho }}} \in {{\bar{\mu }}}(D_{12} \times D_{21})$, the function

54 $$\begin{aligned} {{\bar{z}}}_{\xi } :s \mapsto {{\bar{z}}}({\lambda }+ s \xi ; {{\bar{\rho }}}) \end{aligned}$$

attains its unique minimum in the open interval

55 $$\begin{aligned} {{\bar{I}}}(\xi , {\lambda }):= (- {{\bar{s}}}_\mathrm{{b}}(- \xi , {\lambda }), {{\bar{s}}}_\mathrm{{b}}(\xi , {\lambda })), \end{aligned}$$

where

56 $$\begin{aligned} {{\bar{s}}}_\mathrm{{b}}(\xi , \lambda )= \sup \lbrace s :\lambda ^1 + s \xi ^1, \lambda ^2 + s \xi ^2 \in \varLambda \rbrace . \end{aligned}$$

#### Proof

We follow the arguments of the proof of Lemma 2. The fact that ${{\bar{z}}}$ is strictly convex and differentiable with respect to $\lambda$ implies that ${{\bar{z}}}_\xi$ is strictly convex and differentiable with respect to *s*. Hence ${{\bar{z}}}_\xi$ attains a unique minimum on the closure of ${{\bar{I}}}(\xi , {\lambda })$.

We therefore need only show that ${{\bar{z}}}_{\xi }$ cannot attain its minimum on the boundary of ${\bar{I}}(\xi , {\lambda })$.

Suppose first that ${\bar{s}}_\mathrm{{b}}(\xi , \lambda ) < \infty$. By Fatou’s Lemma,

57 $$\begin{aligned}&\lim _{s \rightarrow {{\bar{s}}}_\mathrm{{b}}(\xi , {\lambda })} \left\{ \int \nu _{12} \exp ^1_{{\lambda ^1} + s \xi ^1} dv + \int \nu _{21}\exp ^2_{{\lambda ^2} + s \xi ^2} dv \right\} \nonumber \\&\quad \ge \left\{ \int \nu _{12} \exp ^1_{{\lambda ^1} + {{\bar{s}}}_\mathrm{{b}}(\xi , {\lambda }) \xi ^1} dv + \int \nu _{21}\exp ^2_{{\lambda ^2} + s_\mathrm{{b}}(\xi , {\lambda }) \xi ^2} dv \right\} dv \end{aligned}$$

Assumption 1 implies that ${\lambda ^1} + {{\bar{s}}}_\mathrm{{b}}(\xi , {\lambda }) \xi ^1 \not \in \varLambda$ or ${\lambda ^2} + {{\bar{s}}}_\mathrm{{b}}(\xi , {\lambda }) \xi ^2 \not \in \varLambda$. Hence at least one of the integrals on the right-hand side above is $\infty$, which implies

58 $$\begin{aligned} \lim _{s \rightarrow {{\bar{s}}}_\mathrm{{b}}(\xi , \lambda )} z_{\xi }(s) = \mu ^{12}_0(\exp ^1_{\lambda ^1}) + \mu ^{21}_0(\exp ^2_{\lambda ^2}) - \lambda \cdot {{\bar{\rho }}} = \infty . \end{aligned}$$

Now suppose instead that ${{\bar{s}}}_\mathrm{{b}}(\xi , \lambda )= \infty$. There are two cases:

**Case 1:** $\xi ^{1} \cdot a^1(v) \le 0$ and $\xi ^{2} \cdot a^2(v) \le 0$ for a.e $v \in {\mathbb {R}}^3$.

Since ${{\bar{\rho }}} \in {\bar{\mu }} (D_{12} \times D_{21})$, there exist $g_1, g_2 \in D_{12} \times D_{21}$ such that ${\bar{\rho }} = {\bar{\mu }}(g_1,g_2)$; that is

59 $$\begin{aligned} {\bar{\rho }} = {\bar{\mu }}(g_1,g_2) = \begin{pmatrix} \mu _0^{12}(g_1) \\ \mu _0^{21}(g_2) \\ \mu _1^{12}(g_1) + \mu _1^{21}(g_2) \\ \mu _2^{12}(g_1) + \mu _2^{21}(g_2) \end{pmatrix}. \end{aligned}$$

By definition, $g_1$ and $g_2$ are not identically zero, and by Assumption 1, $\nu _{kj}>0$. Thus the sets

60 $$\begin{aligned} \varOmega _1&:= \lbrace v \in {\mathbb {R}}^3 \mid \xi ^{1} \cdot a^1(v)<0\rbrace \cap \lbrace v \in {\mathbb {R}}^3 \mid \nu _{12}(v) g_1(v) >0 \rbrace \quad \text {and}\end{aligned}$$

61 $$\begin{aligned} \varOmega _2&:= \lbrace v \in {\mathbb {R}}^3 \mid \xi ^{2} \cdot a^2(v) <0 \rbrace \cap \lbrace v \in {\mathbb {R}}^3 \mid \nu _{21}(v) g_2(v) >0 \rbrace \end{aligned}$$

both have positive measure. Hence

62 $$\begin{aligned} \xi \cdot {\bar{\rho }}&= \xi ^{1} \cdot \mu ^{12}(g_1) + \xi ^{2} \cdot \mu ^{21}(g_2)\end{aligned}$$

63 $$\begin{aligned}&= \int \nu _{12} \xi ^{1} \cdot a^1(v) g_1(v)dv + \int \nu _{21} \xi ^{2} \cdot a^2(v) g_2(v) dv <0, \end{aligned}$$

so that

64 $$\begin{aligned} \lim _{s \rightarrow \infty } {{\bar{z}}}_{\xi }(s)&= \lim _{s \rightarrow \infty } \left\{ \mu ^{12}_0(\exp ^1_{\lambda ^1+s \xi ^1}) + \mu ^{21}_0(\exp ^2_{\lambda ^2 + s \xi ^2}) - (\lambda + s \xi )\cdot {{\bar{\rho }}} \right\} \end{aligned}$$

65 $$\begin{aligned}&> \lim _{s \rightarrow \infty } \left\{ - (\lambda + s \xi ) \cdot {\bar{\rho }} \right\} = \infty . \end{aligned}$$

**Case 2:** The set $\lbrace v \in {\mathbb {R}}^3 \mid \xi ^{1} \cdot a^1(v) >0\rbrace$ or $\lbrace v \in \varOmega \mid \xi ^{2} \cdot a^2(v)>0 \rbrace$ has positive measure.

Without loss of generality, assume that $\lbrace v \in {\mathbb {R}}^3 \mid \xi ^{1} \cdot a^1(v) >0\rbrace$ has positive measure. Then, there exists some $\varepsilon >0$ such that $B=\lbrace v \in {\mathbb {R}}^3 \mid \xi ^{12}\cdot a^1(v)> \varepsilon \rbrace$ also has positive measure. Hence

66 $$\begin{aligned} \lim _{s \rightarrow \infty } {\bar{z}}_{\xi }(s) \ge \lim _{s \rightarrow \infty } \left( \left( \int _B \nu _{12} \exp ^1_{\lambda ^{1}} dx \right) \exp (s \varepsilon ) - ({\lambda } + s \xi ) \cdot \rho _\mathrm{\mathrm{{mix}}} \right) = \infty . \end{aligned}$$

due to exponential growth in *s*. $\square$

#### Theorem 4

For any ${{\bar{\rho }}} \in {{\bar{\mu }}}(D_{12} \times D_{21})$, the function ${\bar{z}}(\cdot , {{\bar{\rho }}})$ has a unique minimizer $\lambda ^* \in {{\bar{\varLambda }}}$.

The proof of this theorem is analogous to the proof of Theorem 3 in the intra-species case.

#### Corollary 2

Given any $f_1 \in D_{12}$ and $f_2 \in D_{21}$, there exist multipliers $\lambda ^{12}$ and $\lambda ^{21}$ such that $\lambda ^{21}_1 = \lambda ^{12}_1$, $\lambda ^{21}_2 = \lambda ^{12}_2$, and the corresponding functions $M^{12}$ and $M^{21}$ given in (2) solve (4).

#### Proof

Let ${{\bar{\rho }}}= {{\bar{\mu }}}(f_1,f_2)$. According to Theorem 4, ${{\bar{z}}}(\cdot , {{\bar{\rho }}})$ has a unique minimizer, which we denote by $\lambda ^*=((\lambda ^*)_0^{1},(\lambda ^*)_0^{2}, (\lambda ^*)_1, (\lambda ^*)_2).$ By Lemma 1, ${{\bar{z}}}(\cdot , {{\bar{\rho }}})$ is also differentiable, so the first-order optimality condition (52) implies that ${{\bar{\rho }}}= {\bar{\mu }}(\exp ^1_{(\lambda ^*)^{1}}, \exp ^2_{(\lambda ^*)^{2}})$. The result then follows from Theorem 2. Finally, we set

67 $$\begin{aligned} \lambda ^{12} = ((\lambda ^*)_0^{1}, (\lambda ^*)_1, (\lambda ^*)_2) \quad \text {and} \quad \lambda ^{21} = ((\lambda ^*)_0^{2}, (\lambda ^*)_1, (\lambda ^*)_2) \end{aligned}$$

and define $M^{12}$ and $M^{21}$ according to (2). $\square$

## Consistency of the Model

The conditions (3) and (4) lead to standard conservation laws and an entropy dissipation statement. We recall a few definitions:

### Definition 2

The mass density, momentum, and energy of an integrable distribution $g = g(v)$ of particles with mass *m* are given by the moments

68 $$\begin{aligned} \rho _g = \int m g(v) dv, \quad q_g = \int m v g(v) dv, \quad \text {and} \quad E_g = \frac{1}{2} \int m |v|^2 g(v) dv, \end{aligned}$$

respectively. The associated mean velocity and temperature are given by

69 $$\begin{aligned} u_g = \frac{q_g}{\rho _g} = \frac{\int v g(v) dv}{\int g(v) dv} \quad \text {and} \quad T_g = \frac{2}{3}\frac{E_g}{\rho _g/m} - \frac{1}{3} \frac{|q_g|^2}{\rho _g} = \frac{1}{3}\frac{\int m |v-u_g|^2 g(v) dv}{\int g(v) dv}. \end{aligned}$$

### Conservation Properties

An immediate consequence of (3) and (4) is the following.

#### Theorem 5

(Conservation of the number of each species, total momentum and total energy) The space-homogeneous form of (1) satisfies

70 $$\begin{aligned} \partial _t \rho _{f_1} = \partial _t \rho _{f_2} =0, \quad \partial _t \left( q_{f_1} + q_{f_2} \right) = 0 , \quad \partial _t \left( E_{f_1} + E_{f_2} \right) = 0 \end{aligned}$$

### Entropy Dissipation and the Structure of Equilibria

Define the total entropy density

71 $$\begin{aligned} H(g_1,g_2) = \int h(g_1)dv + \int h(g_2) dv \end{aligned}$$

and the dissipation density

72 $$\begin{aligned} S(g_1,g_2)&= S_{11}(g_1) + S_{12}(g_1,g_2) + S_{21}(g_1,g_2) + S_{22}(g_2) \end{aligned}$$

73 $$\begin{aligned}&=\int \nu _{11} \ln g_1 ( M_{11} - g_1) dv + \int \nu _{12} \ln g_1 ( M_{12}- g_1) dv \end{aligned}$$

74 $$\begin{aligned}&\quad + \int \nu _{21} \ln g_2 ( M_{21}- g_2) dv + \int \nu _{22} \ln g_2 ( M_{22}- g_2) dv \end{aligned}$$

#### Theorem 6

Assume $g_1,g_2>0$. Then $S(g_1,g_2) \ge 0$ with equality if and only if $g_1$ and $g_2$ are two Maxwellian distributions with equal mean velocity and temperature.

#### Proof

In [29], it is shown that $S_{kk}(g) \ge 0$ with equality if and only if *g* is a Maxwellian. Thus it remains to show a similar result for the combined quantity $S_{12}(g_1,g_2) + S_{21}(g_1,g_2)$. We begin with the following claim:

75 $$\begin{aligned} I(g_1,g_2):= \int \nu _{12} \ln M_{12} ( M_{12} - g_1) dv + \int \nu _{21} \ln M_{21} ( M_{21} - g_2) dv = 0. \end{aligned}$$

Indeed an explicit calculation gives

76 $$\begin{aligned} \ln M_{12} = m_1 \lambda _0^{12} + m_1 \lambda _1\cdot v + m_1 \lambda _2 |v|^2 \quad \text {and} \quad \ln M_{21} = m_2 \lambda _0^{21} + m_2 \lambda _1 \cdot v + m_2 \lambda _2 |v|^2, \end{aligned}$$

which when substituted into (75) gives

77 $$\begin{aligned} I(g_1,g_2)&= \int \nu _{12} ( m_1 \lambda _0^{12}+ m_1 \lambda _1\cdot v + m_1 \lambda _2 |v|^2) ( M_{12} - g_1) dv \end{aligned}$$

78 $$\begin{aligned}&\quad + \int \nu _{21} ( m_2 \lambda _0^{21}+ m_2 \lambda _1\cdot v + m_2 \lambda _2 |v|^2) ( M_{21} - g_2) dv = 0, \end{aligned}$$

due to the constraints (4). From (75), it follows that

79 $$\begin{aligned} \begin{aligned} S_{12}(g_1,g_2)&+ S_{21}(g_1,g_2) = S_{12}(g_1,g_2) + S_{21}(g_1,g_2) - I(g_1,g_2) \\&= \int \nu _{12} \ln \left( \frac{g_1}{M_{12}} \right) ( M_{12} - g_1) dv + \int \nu _{21} \ln \left( \frac{g_2}{M_{21}} \right) ( M_{21} -g_2) dv \\&\le 0. \end{aligned} \end{aligned}$$

with equality if and only if $g_1=M_{12}$ and $g_2=M_{21}$. Moreover, a direct calculation shows that the functions $M_{12}$ and $M_{21}$ have the same mean velocity and temperature:

80 $$\begin{aligned} u_{M_{12}} = u_{M_{21}} =- \frac{\lambda _1}{\lambda _2} \quad \text {and} \quad T_{M_{12}} = T_{M_{21}} =- \frac{1}{2 \lambda _2} \end{aligned}$$

$$\square $$

#### Corollary 3

(Entropy inequality for mixtures) Assume that $f_1, f_2 >0$ are a solution to (1) where the target Maxwellians have the shape (2), then we have the following entropy inequality

81 $$\begin{aligned} \partial _t \left( H(f_1,f_2) \right) + \nabla _x \cdot \left( \int v (h(f_1) + h(f_2)) dv \right) \le 0 \end{aligned}$$

with equality if and only if $f_1$ and $f_2$ are two Maxwellian distributions with equal mean velocity and temperature.

#### Proof

A direct calculation with (1) gives

82 $$\begin{aligned} \partial _t H(f_1,f_2) + \nabla _x \cdot \int (h(f_1)+ h(f_2)) v dv = S(f_1,f_2). \end{aligned}$$

The result then follows immediately from the previous theorem.$\square$

## The *N*-Species Case

The two-species case can be extended to a system of *N*-species that undergo binary collisions. We consider the *N*-species kinetic equation,

83 $$\begin{aligned} \partial _t f_i + v \cdot \nabla _x f_i = \sum _{j=1}^N \nu _{ij} (M_{ij}- f_i), \quad i=1,\ldots ,N. \end{aligned}$$

The quantity $\nu _{ii}$ is the collision frequency of particles of species *i* with itself whereas $\nu _{ij}$ is the collision frequency of particles of species *i* with species *j*, with $i,j=1,\ldots ,N, ~ i \ne j$. We only have terms of this form and not terms containing indices of more than two species because we consider only binary interactions.

For fixed $i,j\in \{1,\dots ,N\}$ the target Maxwellians $M_{ii}$, $M_{jj}$, $M_{ij}$ and $M_{ji}$ are given by (2). The single species target Maxwellians $M_{ii}$ and $M_{jj}$ will be determined such that they satisfy (3). The functions $M_{ij}$ and $M_{ji}$ will be determined such that we obtain conservation of mass of each species and conservation of total momentum and total energy in interactions between these two species, i.e.,

84 $$\begin{aligned} \begin{aligned}&\int \nu _{ij} M_{ij} dv = \int \nu _{ij} f_i dv, \quad \int \nu _{ji} M_{ji} dv = \int \nu _{ji} f_j dv \\&\int \nu _{ij} \begin{pmatrix} m_i v \\ m_i |v|^2 \end{pmatrix} (M_{ij} - f_i) dv = - \int \nu _{ji} \begin{pmatrix} m_j v \\ m_j |v|^2 \end{pmatrix} (M_{ji} - f_j) dv. \end{aligned} \end{aligned}$$

as an obvious generalization of (4). All the proofs concerning existence and uniqueness of the target Maxwellians and the H-Theorem can be proven exactly in the same way as for two species. For the total entropy $H(f_1,\ldots ,f_N) = \int (h(f_1) + \dots + h(f_N)) dv$ we obtain

85 $$\begin{aligned} \partial _t \left( H(f_1,\ldots , f_N) \right) + \nabla _x \cdot \left( \int v (h(f_1) + \dots + h(f_N)) dv \right) \le 0. \end{aligned}$$

## Conclusion

We have presented a multi-species BGK model in which the collision frequencies depend on the microscopic velocity. The model is formally derived based on an entropy minimization principle, which implies that the target functions take the form of Maxwellians. However, contrary to classical BGK models with velocity-independent frequencies, the relationship between the Maxwellian parameters and the moments of the distribution function is not analytic. Thus some effort is required to establish rigorously the existence of parameters which satisfy first-order optimality conditions. We also show that the derived model satisfies an H-Theorem and that it can be extended to the case of arbitrarily many species undergoing binary collisions.

In future work, we will develop numerical tools for discretizing the model developed here, including the numerical solution of the defining optimization problem. A numerical code will enable computational explorations about how to choose the collision frequencies and what benefit is providing by their flexibility. Also, because the motivation for the model is the simulation of multi-species plasmas, we will extend it for use in such contexts by adding self-consistent fields.
